# Supplementary material for: No Association between Glucocorticoid Diurnal Rhythm in Breastmilk and Infant Body Composition at 3 Months
Source: Nutrients. 2019 Oct 2;11(10):2351. doi: 10.3390/nu11102351 (PMC6835896; doi:10.3390/nu11102351)
Supplement: Supplementary file 1 [file nutrients-11-02351-s001.pdf]

**Supplementary Table 1**: Cortisol and cortisone concentrations in breastmilk in 4-hour intervals.		
	Cortisol (nmol/L)	Cortisone (nmol/L)
0:00-4:00	4.1±5.5	15.1±11.8
4:00-8:00	11.6±8.7	29.7±12.8
8:00-12:00	8.2±6.5	27.9±8.6
12:00-16:00	4.4±2.9	21.3±6.8
16:00-20:00	2.1±1.4	13.0±6.1
20:00-24:00	2.1±3.9	10.4±9.2
Values represent mean±SD		
